# Extracellular Vesicles from Aspergillus flavus Induce M1 Polarization *In Vitro*

**DOI:** 10.1128/mSphere.00190-20

**Published:** 2020-05-06

**Authors:** Verônica S. Brauer, André M. Pessoni, Tamires A. Bitencourt, Renato G. de Paula, Liliana de Oliveira Rocha, Gustavo H. Goldman, Fausto Almeida

**Affiliations:** a Department of Biochemistry and Immunology, Ribeirao Preto Medical School, University of Sao Paulo, Ribeirao Preto, Sao Paulo, Brazil; b Department of Physiological Sciences, Health Sciences Centre, Federal University of Espirito Santo, Vitoria, Espírito Santo, Brazil; c Department of Food Science, Faculty of Food Engineering, Campinas State University (UNICAMP), Campinas, Sao Paulo, Brazil; d Faculdade de Ciências Farmacêuticas de Ribeirão Preto, University of Sao Paulo, Ribeirao Preto, Sao Paulo, Brazil; University of Georgia

**Keywords:** *Aspergillus flavus*, extracellular vesicles, macrophage, cytokine, phagocytosis, killing, polarization, *Galleria mellonella*

## Abstract

Aspergillus flavus, a ubiquitous and saprophytic fungus, is the second most common cause of aspergillosis worldwide. Several mechanisms contribute to the establishment of the fungal infection. Extracellular vesicles (EVs) have been described as “virulence factor delivery bags” in several fungal species, demonstrating a crucial role during the infection. In this study, we evaluated production of A. flavus EVs and their immunomodulatory functions. We verified that A. flavus EVs induce macrophages to produce inflammatory mediators, such as nitric oxide, tumor necrosis factor alpha (TNF-α), interleukin-6 (IL-6), and IL-1β. Furthermore, the A. flavus EVs enhance phagocytosis and killing by macrophages and induce M1 macrophage polarization *in vitro*. In addition, a prior inoculation of A. flavus EVs in Galleria mellonella larvae resulted in a protective effect against the fungal infection. Our findings suggest that A. flavus EVs are biologically active and affect the interaction between A. flavus and host immune cells, priming the innate immune system to eliminate the fungal infection. Collectively, our results suggest that A. flavus EVs play a crucial role in aspergillosis.

**IMPORTANCE** Immunocompromised patients are susceptible to several fungal infections. The genus *Aspergillus* can cause increased morbidity and mortality. Developing new therapies is essential to understand the fungal biology mechanisms. Fungal EVs carry important virulence factors, thus playing pivotal roles in fungal pathophysiology. No study to date has reported EV production by Aspergillus flavus, a fungus considered to be the second most common cause of aspergillosis and relevant food contaminator found worldwide. In this study, we produced A. flavus EVs and evaluated the *in vitro* immunomodulatory effects of EVs on bone marrow-derived macrophages (BMDMs) and *in vivo* effects in a Galleria mellonella model.

## Abstract (precis)

Immunocompromised patients are susceptible to several fungal infections. The genus *Aspergillus* can cause increased morbidity and mortality. Developing new therapies is essential to understand the fungal biology mechanisms. Fungal EVs carry important virulence factors, thus playing pivotal roles in fungal pathophysiology. No study to date has reported EV production by Aspergillus flavus, a fungus considered to be the second most common cause of aspergillosis and relevant food contaminator found worldwide. In this study, we produced A. flavus EVs and evaluated the *in vitro* immunomodulatory effects of EVs on bone marrow-derived macrophages (BMDMs) and *in vivo* effects in a Galleria mellonella model.

## INTRODUCTION

Aspergillus flavus is a ubiquitous and saprophytic fungus (1). It is the second most common cause of invasive and noninvasive aspergillosis (infection caused by *Aspergillus* species) (2, 3). Furthermore, A. flavus can contaminate several crops, such as maize, peanuts, and cottonseed, both pre- and postharvest and can cause huge economic losses (2, 4). Ingestion of the fungus-contaminated food can be fatal, mainly due to the mycotoxin production; for example, aflatoxin B1 is a natural carcinogenic compound that affects the liver, resulting in hepatocellular carcinoma, and causes acute aflatoxicosis (5, 6). Immunocompetent individuals rarely suffer from aspergillosis. Nevertheless, in immunocompromised hosts (such as patients with neutropenia or receiving cytotoxic and corticosteroid therapy, undergoing transplantation, and suffering from HIV [human immunodeficiency virus] infection) (8), the aspirated conidia may germinate and colonize the tissue, resulting in the clinical effects of the disease (9). Recently, A. flavus was ranked among the 10 most highly pathogenic fungi worldwide (10).

Extracellular vesicles (EVs) are structures produced by all life domains; they range in size from 30 to 1,000 nm and are surrounded by a lipid bilayer, carry protein, lipids, polysaccharides, nucleic acids, and pigments, and are crucial in cell communication, physiology, and immunopathogenesis of fungal infections (11–13). Fungal EV cargo may influence the host-parasite relationship during fungal infections (14). In 2007, Rodrigues et al., studying Cryptococcus neoformans, initially reported production of EVs by fungi (15). Since then, fungal EV production has been described in several fungal species, such as Cryptococcus gattii (16), Saccharomyces cerevisiae (17), Alternaria infectoria (18), Paracoccidioides brasiliensis (19), Histoplasma capsulatum (20), Candida albicans (20), Candida parapsilosis (20), Sporothrix schenckii (20), Sporothrix brasiliensis (21), Malassezia sympodialis (22), Pichia fermentans (23), Trichophyton interdigitale (24), Trichoderma reesei (25), Aspergillus fumigatus (26), and others.

Considering the *Aspergillus* species, EV production was reported recently in A. fumigatus (26); however, no study has reported EV production by A. flavus. Thus, the present study aimed to evaluate whether A. flavus produces EVs and, if so, whether these EVs are able to stimulate an immune response of macrophages. Here, we demonstrated EV production from A. flavus and that these EVs induce bone marrow-derived macrophages to produce inflammatory mediators and fungicidal activities against conidia. Furthermore, EVs stimulated the M1 phenotype for macrophage polarization.

## RESULTS

### EVs from A. flavus.

**FIG 1:**
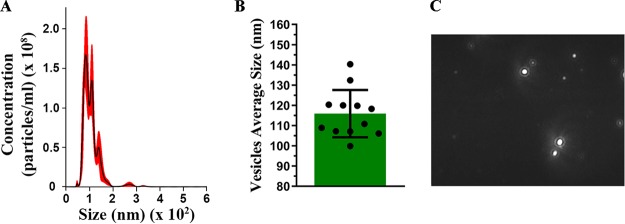
Extracellular vesicles (EVs) produced by Aspergillus flavus. Nanoparticle-tracking analysis of EVs isolated from A. flavus culture supernatant was performed using NanoSight NS300. (A) Representative histogram depicting the particle-size distribution and concentration of EV profiles from A. flavus (EVs × 10^8^ particles/ml). (B) Representative graphic of EV average sizes from 12 independent experiments. (C) Screenshot from the video recorded by NanoSight NS300, presenting EV distribution.

To verify whether A. flavus is able to produce EVs, the supernatant was obtained from conidium culture and EVs were purified. Through nanoparticle tracking analysis (NTA), we determined the size and distribution profile of A. flavus EVs (Fig. 1). These EVs ranged in size from 40 to 400 nm (Fig. 1A), with an average size of 116 nm (±8.35 nm) and median of 114.6 nm from several cultures (Fig. 1). The mode of the diameter of most vesicles was approximately of 83.4 nm. The size and distribution profile of these EVs are illustrated in Fig. 1C via a screenshot of a video recorded by a NanoSight NS300 system.

### A. flavus EVs induce proinflammatory mediators in BMDMs.

**FIG 2:**
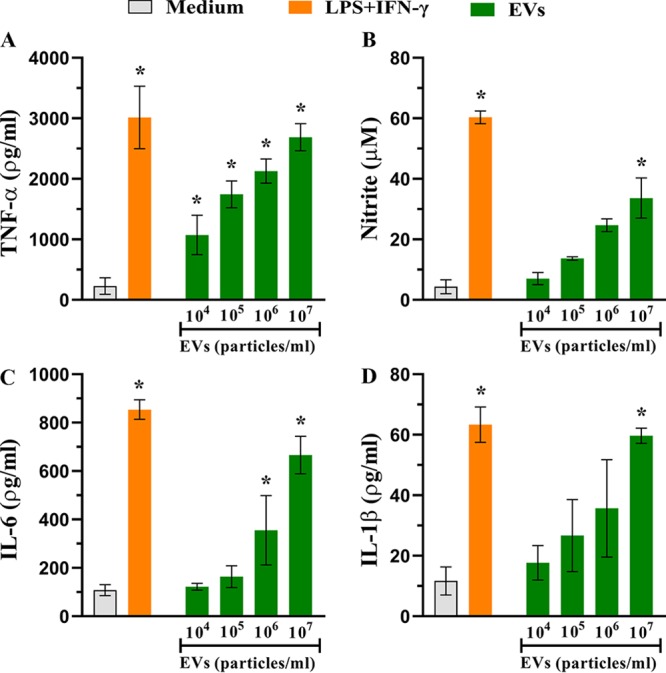
EVs from A. flavus induce the production of inflammatory mediators by bone marrow-derived macrophages (BMDMs). BMDMs obtained from C57BL/6 mice were cultured with different concentrations of EVs (10^4^ to 10^7^ particles/ml) for 48 h at 37°C. As positive and negative controls, the BMDMs were treated with lipopolysaccharide (LPS, 1 μg/ml) plus gamma interferon (IFN-γ) (2 ng/ml) or medium only as indicated. The culture supernatant was used to quantify the levels of (A) tumor necrosis factor alpha (TNF-α), (B) nitrite, (C) interleukin-6 (IL-6), and (D) IL-1β using enzyme-linked immunosorbent assay (ELISA). Data represent results from three independent experiments. One-way ANOVA and Bonferroni’s multiple-comparison tests were used for analysis of TNF-α and IL-6 data, and Kruskal-Wallis and Dunn’s multiple-comparison tests were used for analysis of nitrite and IL-1β data. *, *P* < 0.05.

To evaluate the influence of A. flavus EVs on the host immune response profile, we analyzed whether these EVs stimulated the macrophages. Bone marrow-derived macrophages (BMDMs) were incubated with different concentrations of EVs (10^3^ to 10^7^ particles/ml), with medium alone, or with lipopolysaccharide (LPS) (1 μg/ml) plus gamma interferon (IFN-γ) (2 ng/ml) for 48 h to investigate the production of cytokines and NO. Our findings suggest that A. flavus EVs induce the production of important inflammatory mediators by macrophages (Fig. 2). The production of tumor necrosis factor alpha (TNF-α) (Fig. 2A), NO (Fig. 2B), interleukin-6 (IL-6) (Fig. 2C), and IL-1β (Fig. 2D) occurred in a dose-dependent manner; however, the IL-10 levels in the BMDMs treated with A. flavus EVs were as low as in the nonstimulated cells (data not shown).

### A. flavus EVs enhanced fungicidal activity of macrophages.

**FIG 3:**
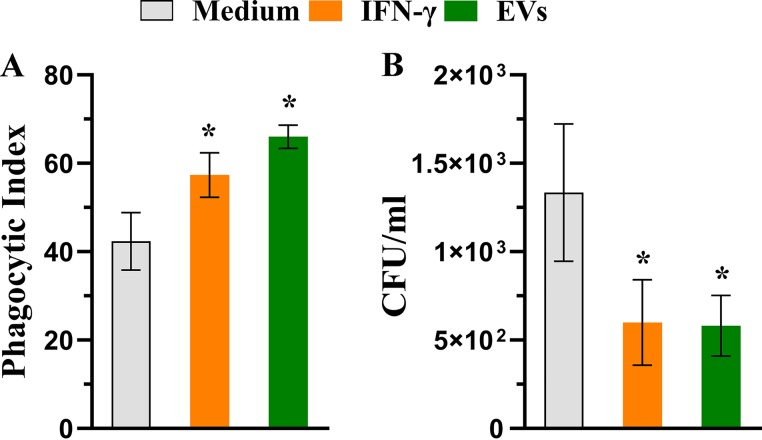
A. flavus EVs stimulate microbicidal activity of BMDMs. (A) BMDMs were plated on glass coverslips and cultured with EVs (10^7^ particles/ml) for 30 min and were treated with A. flavus conidia (macrophages/conidia = 1:1) for 4 h at 37°C, and the phagocytic index was determined. (B) BMDM previously treated with EVs (10^7^ particles/ml), for 30 min, were infected with A. flavus conidia (macrophages/conidia = 1:1) for 48 h at 37°C. The cells were lysed, and the lysate was plated to detect the viable fungi based on CFU counting technique. Data represents results from three independent experiments. For both phagocytosis and killing assays, the IFN-γ-containing medium and medium only were used as positive and negative controls, respectively. An unpaired, two-tailed *t* test was used from both analyses. *, *P* < 0.05.

Since phagocytosis and killing are important effector functions in macrophages (27), we determined the phagocytic index of BMDMs and investigated whether EVs promote A. flavus fungicidal activity by macrophages. As shown in Fig. 3A, BMDMs stimulated with EVs presented enhanced conidium engulfment (55.6% more conidia) compared with the cells cultured only with the medium. This percentage was also higher than the positive-control IFN-γ, with a 35.2% increase compared to the medium condition. Furthermore, we evaluated the fungicidal activity by using killing assays. We performed the phagocytosis assay, and additionally, we treated BMDMs with fungal conidia (ratio of macrophages/conidia = 1:1) for 48 h. The lysate was plated, and the viable fungal cells were enumerated. As depicted in Fig. 3B, the CFU rates from lysate BMDMs stimulated with either IFN-γ or EVs were lower than those seen under the medium condition. These results suggest that A. flavus EVs promote the uptake of A. flavus and enhance the fungicidal activity of BMDMs.

### A. flavus EVs induce the macrophage M1 phenotype.

**FIG 4:**
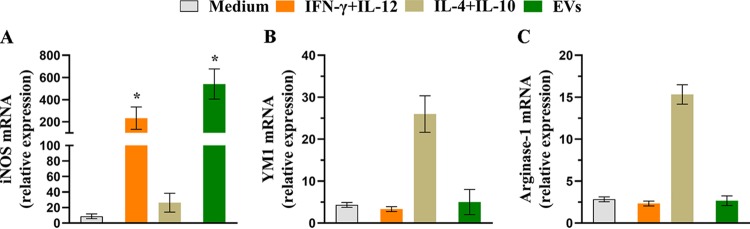
EVs induce M1 polarization of BMDMs. BMDMs were cultured with 10^7^ particles/ml for 6 h. The cells were further treated with IFN-γ (2 ng/ml) plus IL-12p40 (50 ng/ml) as a positive control for M1 phenotype or with IL-4 (50 ng/ml) plus IL-10 (50 ng/ml) as a positive control of M2 phenotype or with medium only as a negative control. The total RNA was extracted from the macrophages and converted into cDNA, and qRT-PCR analysis was performed to evaluate the relative expression levels of classical markers of macrophage polarization. (A) Inducible nitric oxide synthase (iNOS). (B) YM1. (C) Arginase-1. Data represents results from three independent experiments. An unpaired, two-tailed *t* test was used for iNOS, and an unpaired, two-tailed *t* test and Mann-Whitney test were used for YM1 and arginase. *, *P* < 0.05.

BMDMs stimulated with A. flavus EVs produced proinflammatory mediators and enhanced phagocytosis and killing rates. These results suggest macrophage polarization to the “classical activated M1” phenotype. To confirm this hypothesis, BMDMs were treated with EVs (10^7^ particles/ml) or IFN-γ plus IL-12p40 (positive control for M1 polarization), IL-4 plus IL-10 (positive control for M2 polarization), or medium only for 6 h, and quantification of the relative levels of the transcripts of polarization markers (inducible nitric oxide synthase [iNOS], YM1, and arginase-1) was performed. The iNOS (M1 marker) mRNA level was increased by 62-fold in the EV-stimulated BMDMs, representing a response higher than that seen with the positive control (IFN-γ plus IL-12p40) (Fig. 4A). Nevertheless, the mRNA levels of M2 polarization markers (arginase-1 and YM1) seen under conditions of EV stimulation remained close to the mRNA levels of the nonstimulated BMDMs. These data suggest that A. flavus EVs promote BMDM polarization toward the M1 phenotype.

### A. flavus EVs reduce fungal burden in a Galleria mellonella model of infection by A. flavus.

**FIG 5:**
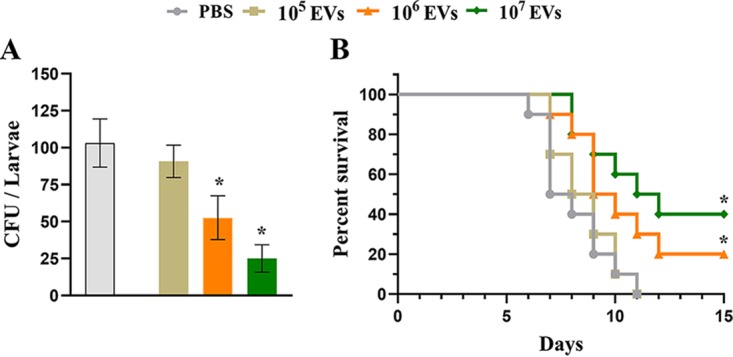
A. flavus EVs induce a protective effect on Galleria mellonella in an *in vivo* model. (A) G. mellonella larvae (*n* = 5 per group) were stimulated with A. flavus EVs (10^5^, 10^6^, and 10^7^ particles) or with PBS as a control 48 h prior to infection with A. flavus conidia. Larvae were homogenized and levels of CFU content determined 48 h postinfection for fungal burden analysis. (B) Survival rates (*n* = 5 larvae per group) during 15 days postinfection. Data represent the results of two independent experiments. One-way ANOVA and Bonferroni’s multiple-comparison tests were used for fungal burden analysis and the log rank (Mantel-Cox) test for survival curve analysis. *, *P* < 0.05.

Since A. flavus EVs were able to stimulate killing activity from BMDMs, we decided to analyze the infection in a G. mellonella *in vivo* model. Prior to challenge, the larvae were treated with different concentrations of EVs (10^5^, 10^6^, and 10^7^) or with phosphate-buffered saline (PBS) as a control. After 48 h, A. flavus conidia were inoculated in the larvae. As shown in Fig. 5A and B, stimulation of EVs resulted in dose-dependently decreased CFU levels and enhanced survival of the larvae, respectively. These results suggest that A. flavus EVs may prime the host innate immune system to eliminate the fungal infection.

## DISCUSSION

This was the first study to demonstrate that A. flavus produce EVs. We have described the production and isolation of A. flavus EVs, the induction of proinflammatory mediators in stimulated macrophages with EVs, and enhanced fungicidal activity. Furthermore, A. flavus EVs increased the transcript levels of iNOS, a classical M1 polarization marker; however, they did not affect the YM1 and arginase-1 levels (M2 polarization markers). We also demonstrated that EVs are biological active using a G. mellonella *in vivo* model, in which prior stimulation of larvae with A. flavus EVs promoted decreased fungal burden and increased survival of larvae during the exposure to A. flavus conidia.

Considering the description of EV production from fungal species, these structures have been considered to be important carriers of biological compounds and are related to several functions such as pathogenicity, cell communication, and immunogenicity of fungal infections (12). Several studies previously described biologically active EVs produced by the fungal species (T. interdigitale, P. brasiliensis, C. neoformans, C. albicans, M. sympodialis, S. brasiliensis) (21, 22, 24, 28–32). Among the *Aspergillus* spp., EVs from A. fumigatus were able to stimulate TNF-α and chemokine (C-C motif) ligand 2 (CCL2) production by macrophages *in vitro* (26).

In accordance with our findings, EVs produced by A. fumigatus (26), T. interdigitale (24), P. brasiliensis (28), and C. albicans (29) were able to increase the effector activity of macrophages in different experimental models. In contrast, EVs from H. capsulatum decreased both the phagocytic activity and killing rate of BMDMs (33), suggesting an immunomodulatory activity of EVs. Depending on the pathogen, the fungal EVs can intensify or attenuate the progression of the infection (14).

Mechanisms involved in the relationship between EVs and immune system remain unclear. EVs carry tremendous molecular content, including molecules that interact with the pattern recognition receptors expressed on cells of the immune system (for example, receptors from family of C-type lectin), thereby resulting in activation of innate and/or adaptive responses and protection of the host against infection (34, 35). Thus, EVs may act as key modulators of the immune response for different fungal infections, thus demonstrating the potential of these structures as possible targets for immune interventions (14).

By inhalation, A. flavus conidia reach the respiratory tract and encounter the innate immune system cells, as alveolar macrophages (36), representing the first line of defense against the pulmonary fungal infection (37). Macrophages contain mechanisms to kill invader pathogens (such as phagocytosis, developed lysosomal compartment, cytokines, chemokines, and growth factor production) (38), as well as mechanisms of antigen processing and presentation in T cells, thus developing an adaptative immune response (39). Macrophages exhibit outstanding phenotypic plasticity to adapt their functions according to their microenvironment (40, 41), thereby altering their phenotype between the classically activated M1 and alternatively activated M2 phenotypes (42). While M2 macrophages are associated with such immunoregulatory functions and markers as inflammatory zone 1 (FIZZ-1), arginase-1, and chitinase-like YM1 (39, 40, 43), M1 macrophages are inflammatory, tumoricidal, and microbicidal, having iNOS nitric oxide synthase 2 as a hallmark molecule (39, 40, 42, 44, 45). The switch between the M1 and M2 phenotypes may represent an important event that defines the progress of fungal infection (46).

During experimental infection by A. flavus, an inflammatory state is established on the mouse lungs, characterized by production of cytokines, including IFN-γ and IL-6, and by recruitment of the immune system cells (neutrophils, lymphocytes, and macrophages); however, at 12 h postinfection, decreased TNF-α levels and higher IL-10 concentrations are observed, suggesting an inflammatory form of regulation (47). Nevertheless, no studies have evaluated the macrophage polarization profile associated with infections by A. flavus. We have provided evidence about the inflammatory profile of macrophages stimulated with EVs, reflected by enhanced inflammatory mediator production and higher levels of phagocytosis and killing activities. Furthermore, the higher level of expression of iNOS and lower level of expression of YM1 and arginase-1 suggest M1 polarization. Similar M1 phenotype polarization results were also described previously in experiments in which BMDMs were stimulated with EVs from T. interdigitale (24). In P. brasiliensis, by reinforcing the M1 polarization, secondary stimulation of macrophages by EVs was able to induce the switch from the M2 phenotype to the M1 phenotype (28). Collectively, these data corroborate the ability of EVs to modulate the immune response and the relationship between the host and pathogen.

The M1 phenotype of macrophages plays a crucial role in fungal elimination. Both M1 development and M2 phenotype inhibition have been described as being protective against A. fumigatus, C. neoformans, and H. capsulatum infections (39, 48, 49). Thus, we hypothesize that M1 polarization stimulated by A. flavus EVs might favor fungal clearance. Taking advantage of the G. mellonella model, which functionally mimics the mammal innate immune system and displays important functions such as phagocytosis and reactive oxygen species production (50, 51), we demonstrated that A. flavus EVs were able to stimulate G. mellonella responses to A. flavus infection. This model had been used previously for pathogenicity analysis of different fungal species, such as A. fumigatus (52), C. albicans (53), and C. neoformans (54), including the effects of fungal EVs in C. albicans and C. neoformans (29, 55). Corroborating our results, prestimulation of G. mellonella with C. albicans EVs was also previously found to result in decreased CFU levels and increased survival of larvae (29). These data suggest that prestimulation of G. mellonella with EVs may prime the immune system and favors the fungal clearance. On the other hand, intramuscular *in vivo* injection in BALB/c mice of EVs from Sporothrix brasiliensis followed by subcutaneous fungal infection resulted in higher fungal burden and larger skin lesions than were seen with mice that did not receive EVs, suggesting that EVs may favor the establishment of *S. brasiliensis* infection (21). Despite the use of the different experimental models, the data collectively corroborate the view that EVs produced by fungal species are bioactive, playing immunomodulatory functions and influencing the pathogenesis of infection.

In this report, we have shown that A. flavus is able to produce and secrete EVs. These structures are immunogenic, stimulate microbicidal functions in macrophages, cause M1 polarization, and have protective effects against A. flavus infection. Our findings provide new evidence on A. flavus infection. EVs produced during the infection, with their immunomodulatory functions, could be suggested to be possible therapeutic targets to treat aspergillosis caused by A. flavus.

## MATERIALS AND METHODS

### Ethics statement.

All experimental procedures using mice followed the Ethical Principles Guide for the Care and Use of Laboratory Animals adopted by the Brazilian College of Animal Experimentation. This study was approved by the Committee of Ethics in Animal Research of the Ribeirao Preto Medical School at the University of Sao Paulo, Brazil (RPMS-USP; protocol 242/2019).

### A. flavus strain and culture conditions.

The A. flavus NRRL 6513 strain was used in all experiments. The strain was maintained in potato dextrose agar (PDA; BD, Sparks, MD, USA) for 5 to 7 days at 30°C for sporulation. For EV production, the conidia were enumerated using a Neubauer chamber, and we inoculated 2 × 10^7^ spores on 400 ml of minimal medium {1% [wt/vol] glucose, 20 ml of a 20× salt solution [120 g/liter NaNO_3,_ 10.4 g/liter KCl, 30 g/liter KH_2_PO_4_, 10.4 g/liter MgSO_4_·7H_2_O], 0.1% [vol/vol] trace elements solution [22 g/liter ZnSO_4_, 11 g/liter H_3_BO_3_, 5 g/liter MnCl_2_, 5 g/liter FeSO_4_, 1.6 g/liter CoCl_2_, 1.6 g/liter CuSO_4_, 1.1 g/liter (NH_4_)_2_MoO_4_, 50 g/liter ethylenediaminetetraacetic acid (EDTA)]} and the pH was adjusted to 6.5 with NaOH. The culture was maintained for 72 h at 200 rpm and 37°C.

### Isolation, characterization, and quantification of EVs.

The culture was isolated as described by Vallejo et al. (19) with slight modifications. After 72 h of culture in the minimal medium, the supernatant was obtained using sterile Miracloth (Millipore, Billerica, MA, USA) and was concentrated via the use of an Amicon ultrafiltration system (Millipore, Billerica, MA, USA) (100-kDa cutoff), centrifuged at 15,000 × *g* for 15 min at 4°C, and ultracentrifuged at 60,000 rpm at 4°C for 1 h. The EVs were suspended in 500 μl of sterile nuclease-free water (Sigma-Aldrich, St. Louis, MO, USA).

The size and distribution of EVs from independent growth cultures were verified via nanoparticle tracking analysis (NTA) using a NanoSight NS300 system (Malvern Instruments, Malvern, United Kingdom) as previously described (24). The particle size, distribution, and quantification were carried out using NanoSight software (version 3.2.16).

### Preparation of BMDMs.

BMDMs were isolated as described by Marim et al. and Bitencourt et al. (24, 56), with slight modifications. Briefly, the bone marrow cells were obtained from the femur and tibia of adult (6 weeks of age), WT (wild-type), male C57BL/6 mice. To release the cells, the bone marrow was flushed with RPMI 1640 medium, and the obtained cells were cultured for 6 days using RPMI 1640 supplemented with 20% fetal cow serum and 30% L-929 cell-conditioned medium (as a source of macrophage/granulocyte colony-stimulating factor). The adherent cells were collected and enumerated using a Neubauer chamber. The BMDMs were plated in the presence of the following different stimuli: EVs (10^3^ to 10^7^ particles/ml), lipopolysaccharide (LPS; 1 μg/ml) plus gamma interferon (IFN-γ) (2 ng/ml) or IFN-γ (2 ng/ml) plus interleukin-12p40 (IL-12p40) (50 ng/ml), IL-4 (50 ng/ml) plus IL-10 (50 ng/ml), or medium only. The cultures were maintained for 6 h for quantitative reverse transcription-PCR (qRT-PCR) analysis or for 48 h for quantification of cytokines and nitric oxide.

### Measurement of nitric oxide (NO) production.

BMDMs were plated on 48-well plates (1.5 × 10^6^ cells/ml; 7.5 × 10^5^ cells/well), and after 48 h, the supernatant was collected and used for NO production. NO production was measured as described by Green et al. (57). Briefly, 50 μl of the cell supernatant was incubated with the same volume of Griess reagent (1.0% sulfanilamide, 0.1% naphthalene diamine dihydrochloride, 2.5% H_3_PO_4_) for 10 min at room temperature. The content was analyzed at a wavelength of 550 nm on a microplate-scanning spectrophotometer (PowerWave-X; BioTek Instruments, Inc., Winooski, VT, USA). With a standard curve generated by using the known concentrations of NaNO_2_ diluted on RPMI medium, the absorbance data were converted into NO concentration values (expressed as micomoles).

### Cytokine measurement.

The cytokine measurement was verified for the BMDM culture supernatant (48 h; 1.5 × 10^6^ cells/ml; 7.5 × 10^5^ cells/well in a 48-well plate). The cytokines (tumor necrosis factor alpha [TNF-α], IL-6, IL-1β, and IL-10) were quantified using an enzyme-linked immunosorbent assay (ELISA) kit, according to the protocol of the manufacturer (BD Biosciences, Pharmingen, San Diego, CA, USA). The concentrations were calculated using standard curves prepared with murine recombinant cytokines, and the sample absorbance was read at 450 nm using a microplate-scanning spectrophotometer (PowerWave-X; BioTek Instruments, Inc., Winooski, VT, USA).

### qRT-PCR analysis.

After 6 h of BMDM culture (2 × 10^6^ cells/ml; 1 × 10^6^ cells/well; 24-well plates), the cells were used to obtain the total RNA content using TRIzol reagent (Invitrogen, Life Technologies, Camarillo, CA, USA), and the protocol was performed in accordance with the manufacturer’s instruction. The RNA was subjected to reverse transcription on cDNA, applying an ImProm-II reverse transcription system (Promega, Fitchburg, WI, USA) using oligo(dT). The qRT-PCR and the primers used were as described by da Silva et al. (28). The transcript levels were analyzed, and β-actin was used as an endogenous control.

### Phagocytosis and killing assay.

To obtain the phagocytic index, 2 × 10^5^ cells/well were plated on glass 13-mm-diameter coverslips placed on 24-well plates (Dulbecco’s modified Eagle medium [DMEM] with 10% fetal bovine serum [FBS]). The cells were treated with IFN-γ (50 ng/ml), EVs (10^7^ vesicles/ml), or medium alone for 30 min. Thereafter, the cells were washed with phosphate-buffered saline (PBS), and BMDMs were treated with conidia of A. flavus (2 × 10^5^ conidia; macrophages/conidia = 1:1) for 4 h at 37°C with 5% CO_2_. Furthermore, the glass coverslips were washed with PBS and stained with Giemsa. An average of 100 macrophages was enumerated to determine the percentage of conidia that were ingested per macrophage.

For the killing assay, 5 × 10^5^ cells/well were plated on a 24-well plate and treated under conditions similar to those described for the phagocytosis assay. Next, the BMDMs were treated with 5 × 10^5^ conidia of A. flavus (macrophages/conidia = 1:1) for 48 h, in DMEM plus 10% FBS, at 37°C and 5% CO_2_. The culture supernatant was discarded after centrifugation (3,500 rpm, 10 min), and the cells were washed with PBS, followed by lysis with cold water. A serial dilution was prepared using the lysate, and the cells were then plated on PDA. The plates were incubated at 37°C for 48 h. The viable fungi were enumerated, and the CFU counts per milliliter were calculated.

### Fungal burden assay and Galleria mellonella survival.

Fungal burden and G. mellonella survival assays were performed as described previously (6, 29, 58) with slight modifications. Briefly, 10 larvae per group were selected that were similar in weight (approximately 275 to 330 mg) and without any gray coloring marks. Then, a 50-μl volume containing 10^5^, 10^6^, or 10^7^ EVs was injected into the last left proleg, directly on hemocoel. PBS was used as a control. The larvae were maintained at 37°C in the dark for 48 h. Then, an A. flavus spore solution (1 × 10^4^ spores/ml) was prepared, and a 10-μl volume was inoculated in all selected larvae, totaling 100 spores/larvae, at the same site of injection. The mortality rate of larvae (*n* = 5) was monitored daily during 15 days; the larvae that did not present movement after touch stimulation were considered dead. Two days postinfection, larvae (*n* = 5) were homogenized on PBS, and the resulting solution was plated on PDA. The plates were incubated at 37°C, for 48 h, and CFU counts were determined.

### Statistical analysis.

The statistical analysis was performed using GraphPad Prism software version 8.0.1 (GraphPad Software, San Diego, CA). The experiments were carried out in triplicate (3 independent experiments), and the graphs present means ± SD (standard deviations). One-way analysis of variance (ANOVA) and Bonferroni’s multiple-comparison tests were used when the data followed a normal distribution, and Kruskal-Wallis and Dunn’s multiple-comparison tests were used for the nonparametric data. In addition, a two-tailed unpaired *t* test was also used for the parametric data and a two-tailed unpaired *t* test along with a Mann-Whitney test for nonparametric data. The *in vivo* experiments were performed in duplicate (two independent experiments), with 5 larvae per group for both experiments (Galleria mellonella survival assay and fungal burden assay). For comparisons of survival curves, log rank (Mantel-Cox) tests were used. For fungal burden, the graphed data are expressed as means ± SD, and the data were compared using one-way ANOVA and Bonferroni’s multiple-comparison tests. *P* values of <0.05 were considered to be statistically significant, and all conditions were compared with the “medium” condition.
